# Supplementary figures and images for: Apiaceae FNS I originated from F3H through tandem gene duplication
Source: PLoS One. 2023 Jan 19;18(1):e0280155. doi: 10.1371/journal.pone.0280155 (PMC9851555; doi:10.1371/journal.pone.0280155)

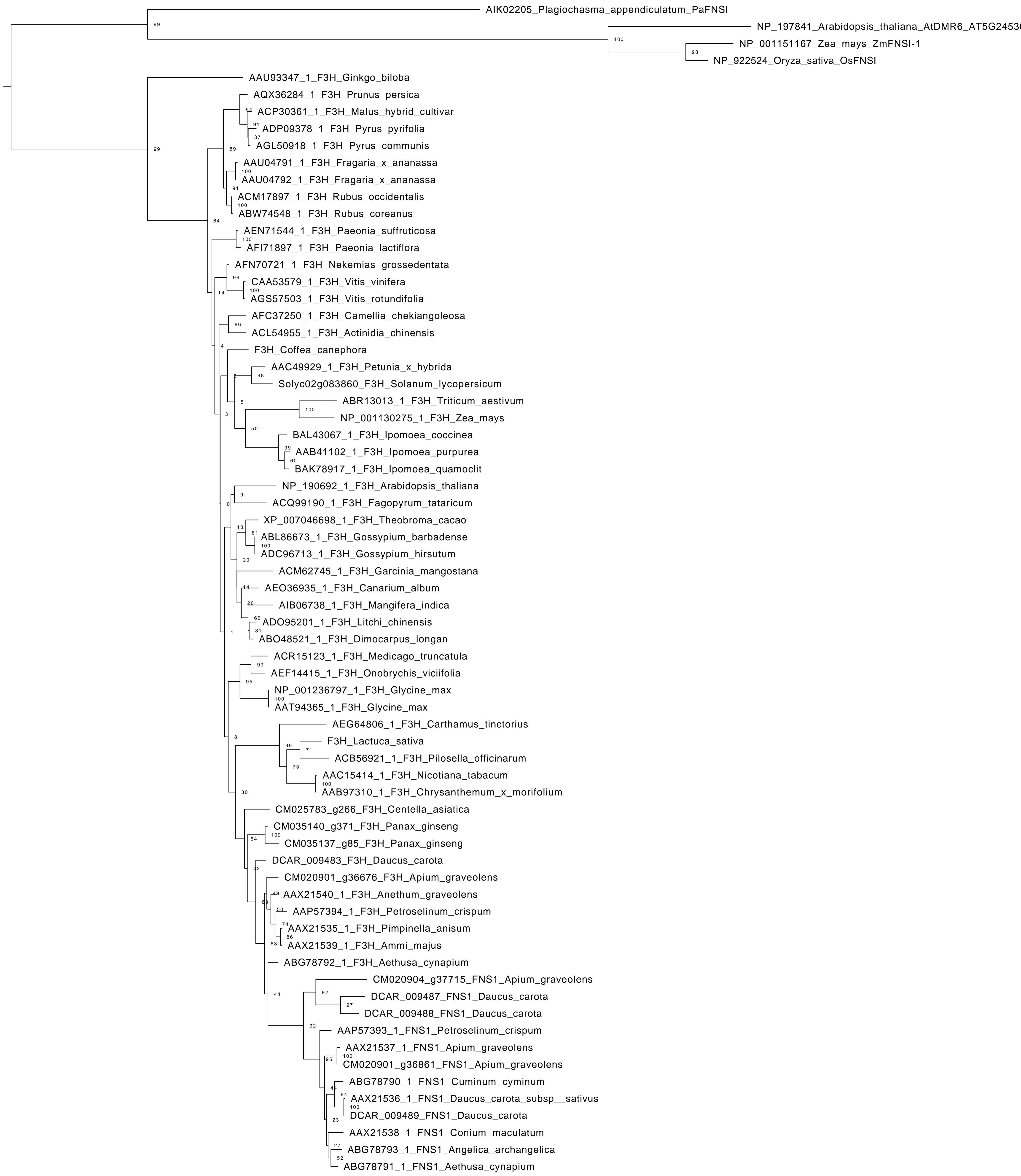

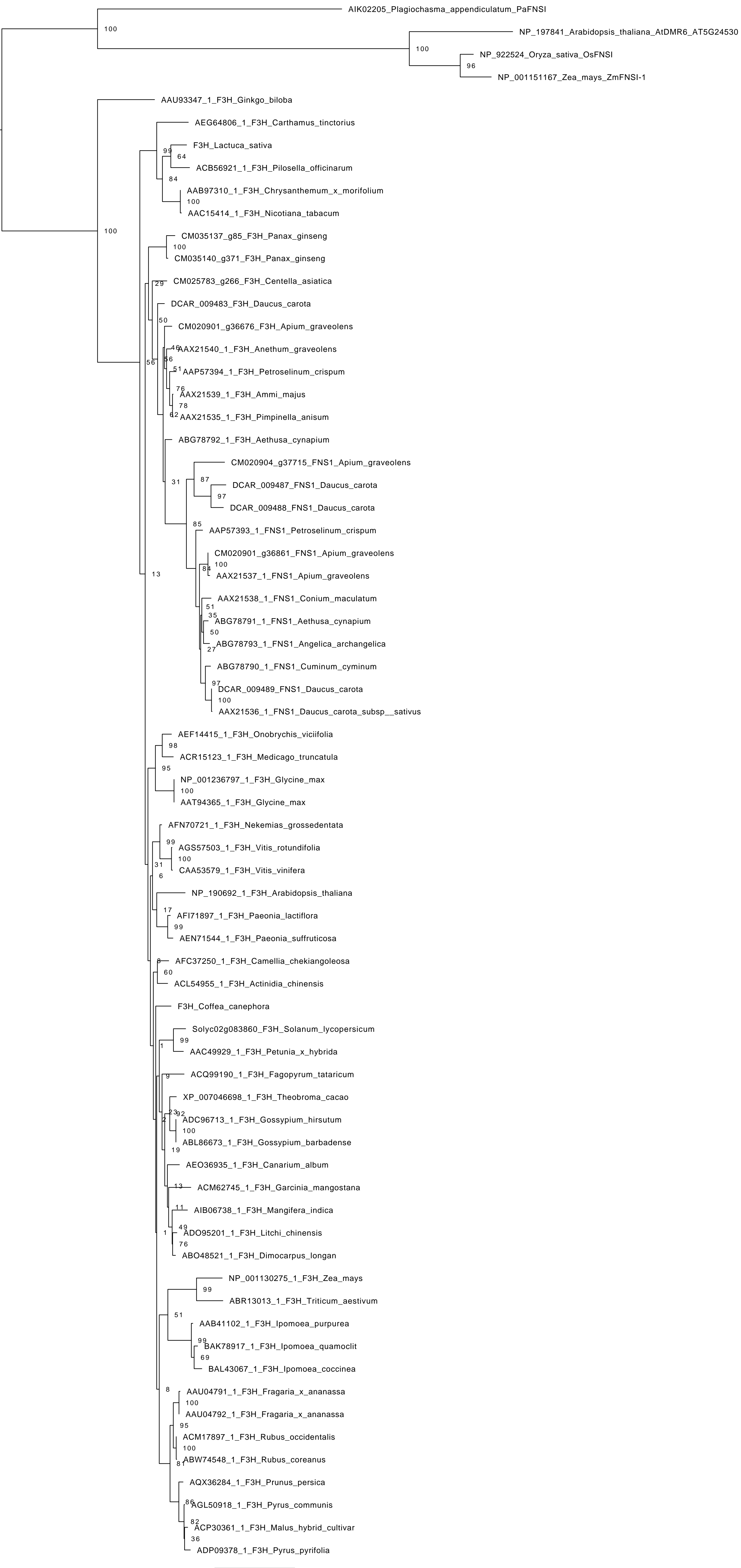

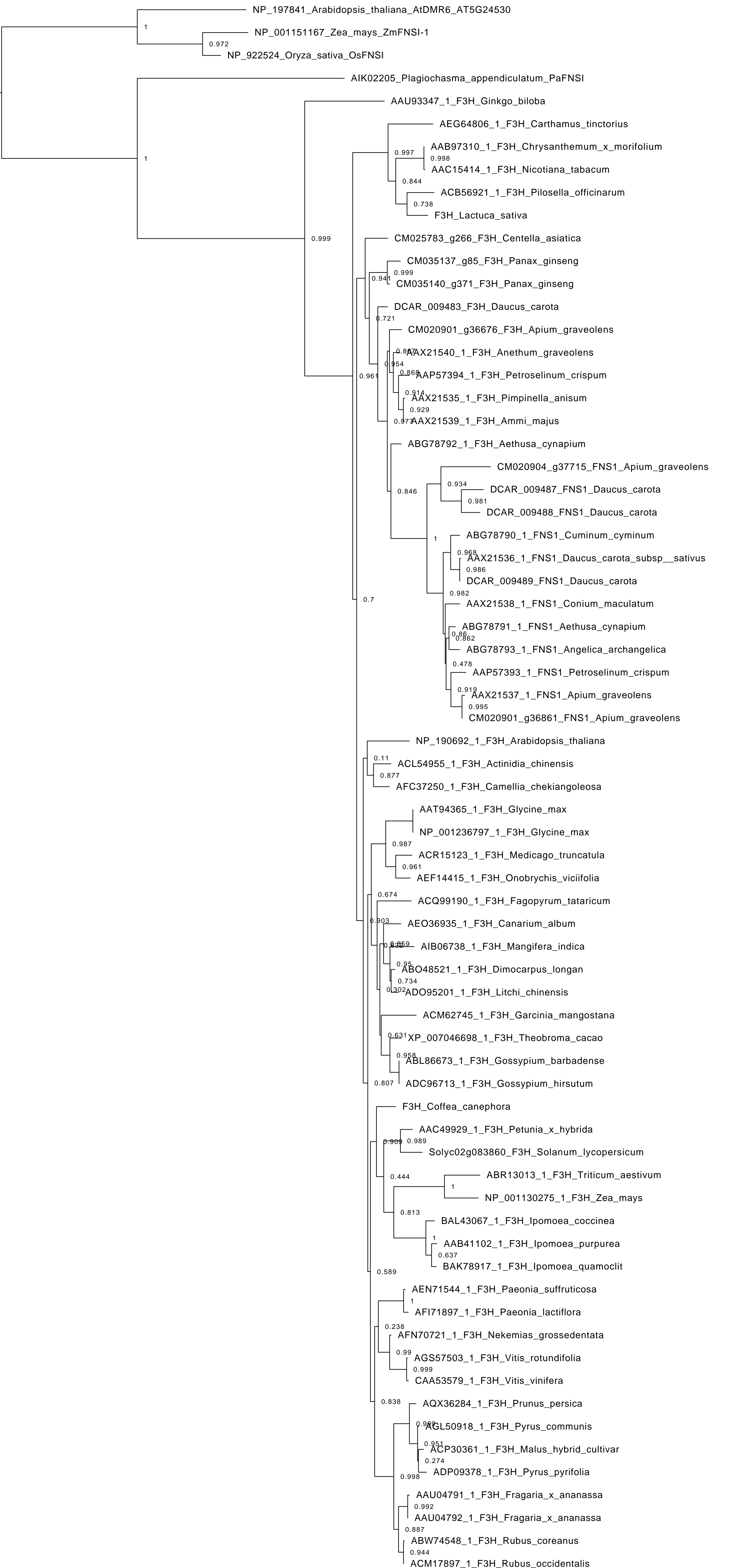

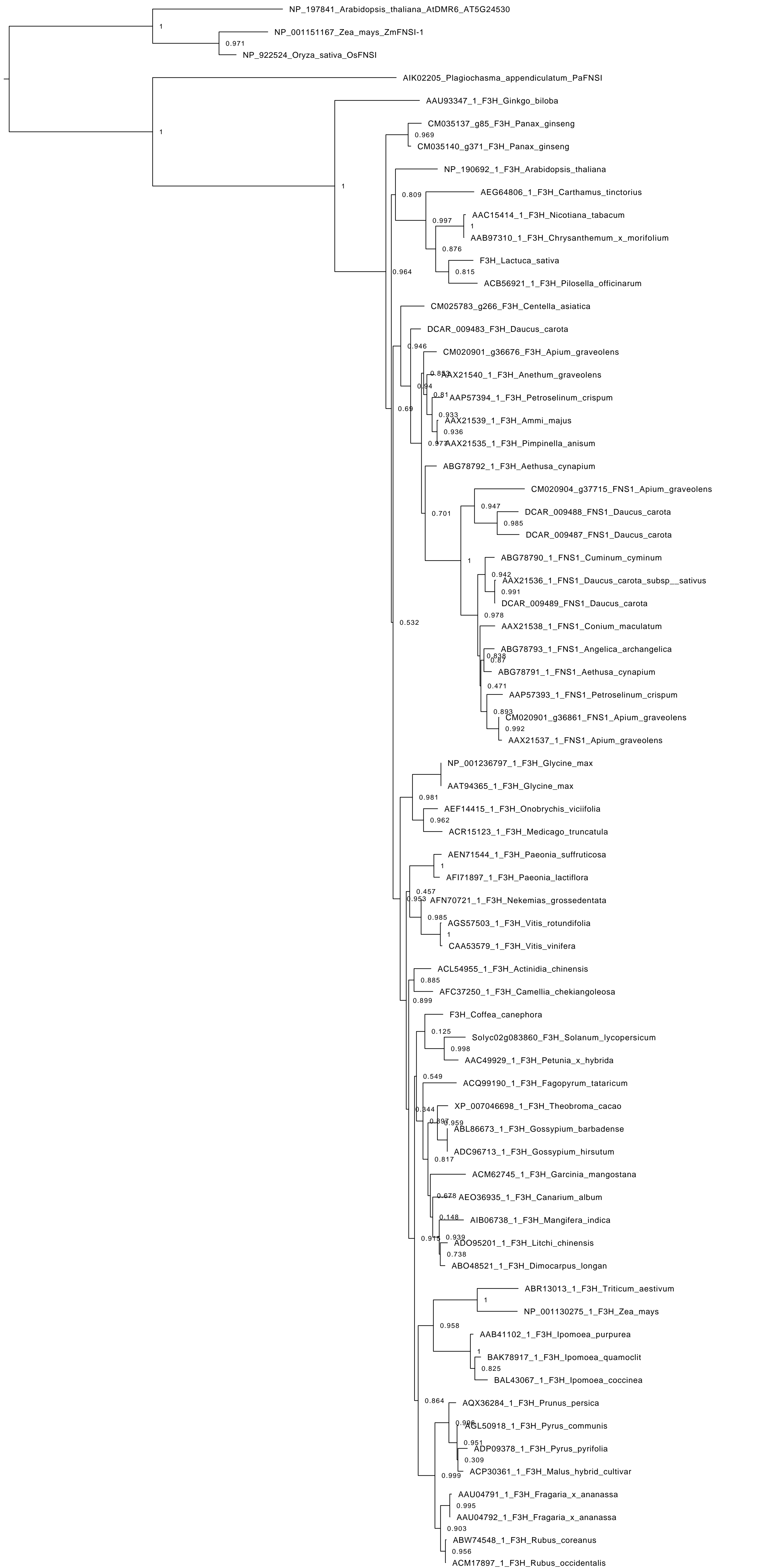

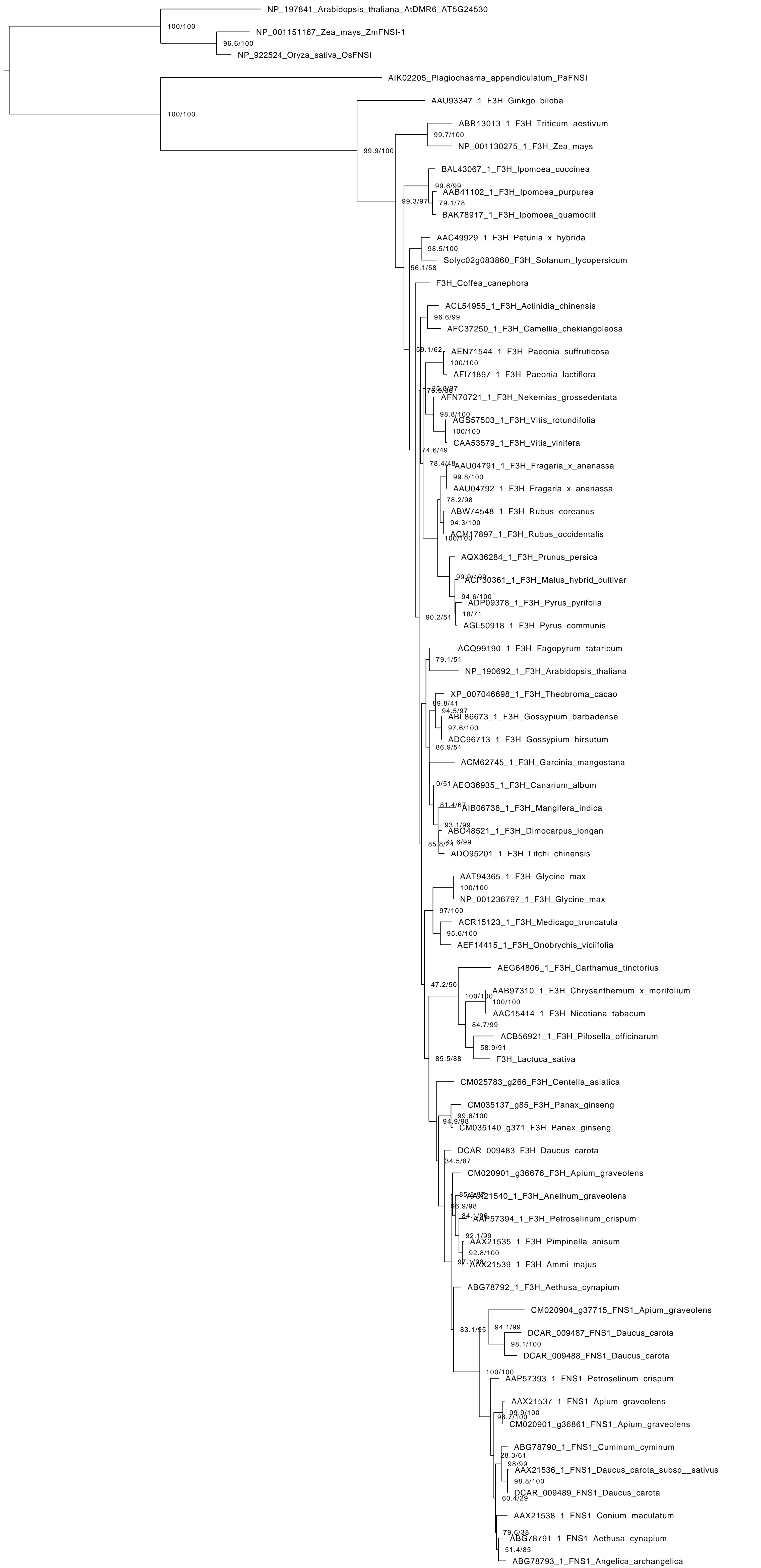

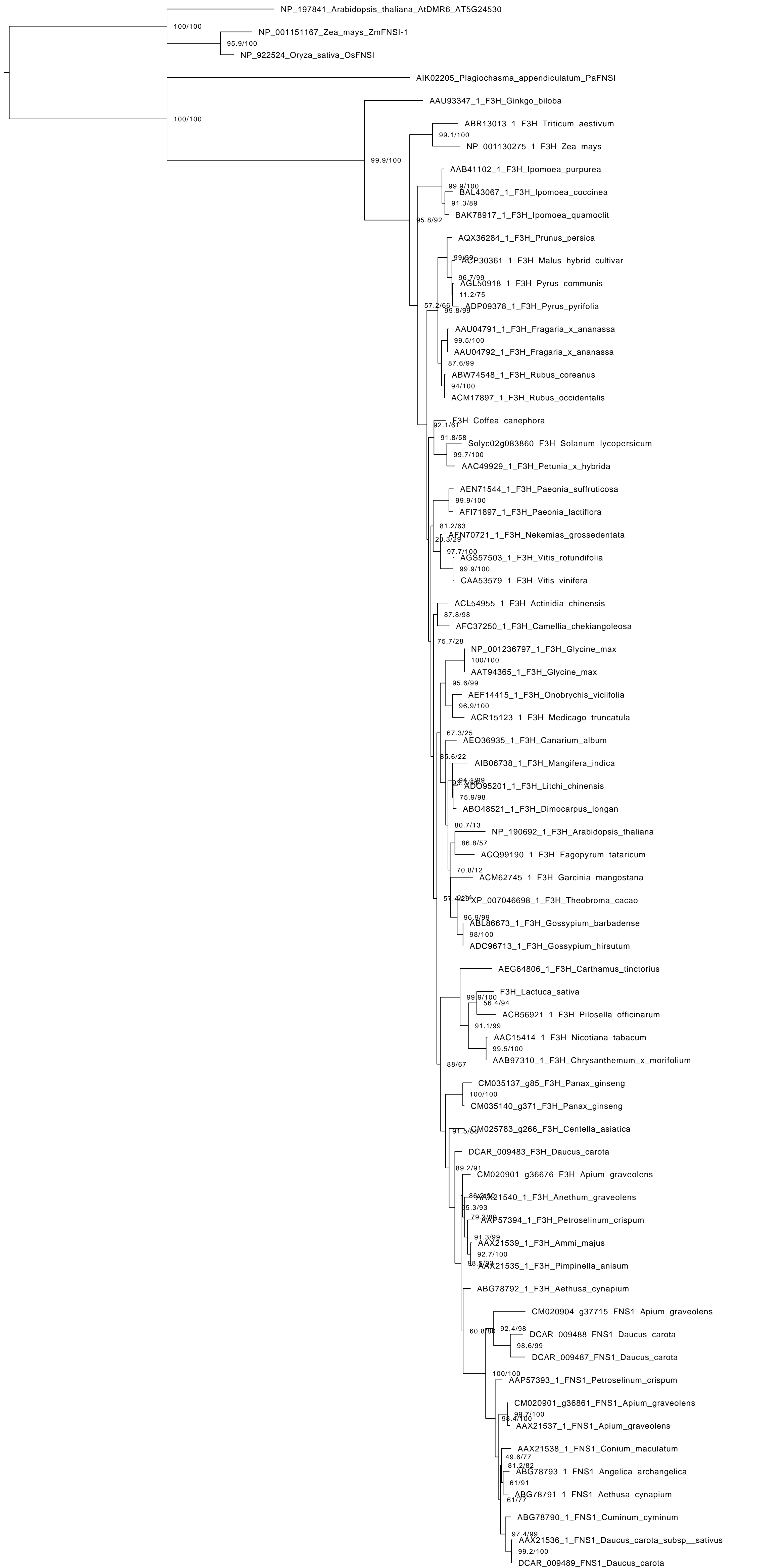

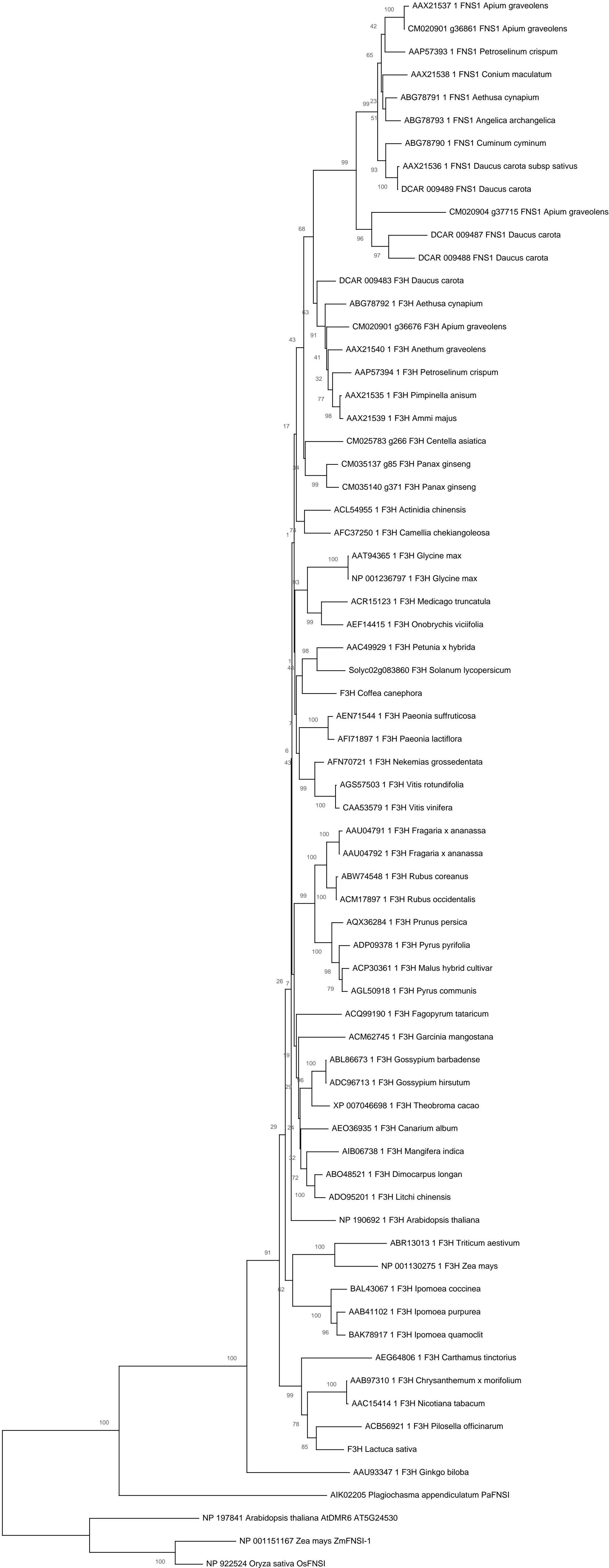

0.10

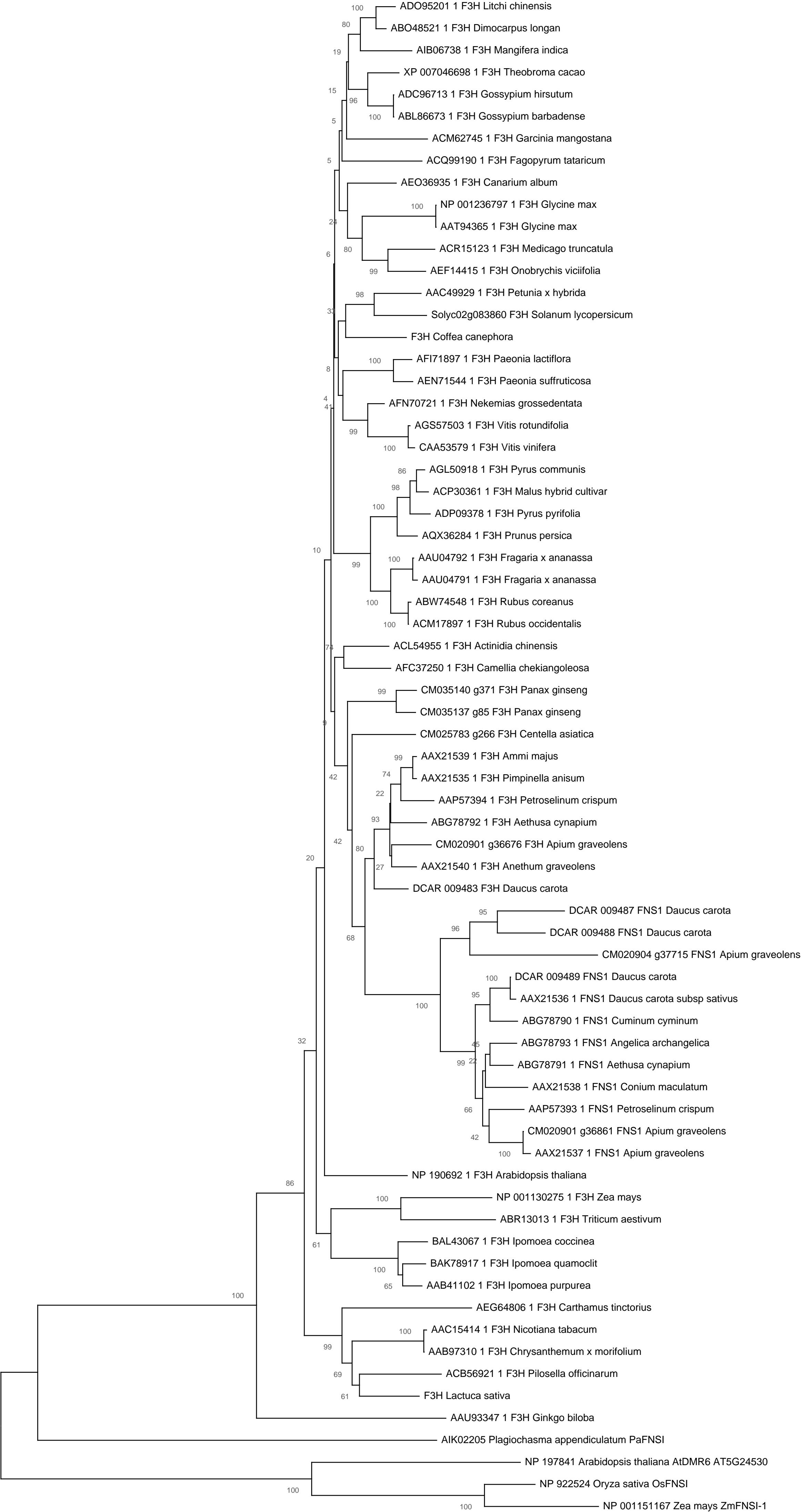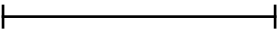

0.10

Supplement: S3 File — (A) Constructed by RAxML based on a MAFFT alignment, (B) constructed by RAxML based on a MUSCLE5 alignment, (C) Constructed by FastTree2 based on a MAFFT alignment, (D) Constructed by FastTree2 based on a MUSCLE5 alignment, (E) constructed by IQ-TREE based on a MAFFT alignment, (F) constructed by IQ-TREE based on a MUSCLE5 alignment, (G) constructed by MEGA based on a MAFFT alignment, and (H) constructed by MEGA based on a MUSCLE5 alignment. (PDF) [file pone.0280155.s003.pdf]
